# Translation, cultural adaptation, and psychometric validation of the simplified Chinese EORTC QLQ-SH22 among cancer patients

**DOI:** 10.1186/s12955-026-02567-z

**Published:** 2026-06-18

**Authors:** Yi-mei Du, Ji Lu, Hong Zhang, Qin Huang, Bing-hua Wang, Li Liu

**Affiliations:** 1 Department of Nursing, Tongji Hospital, Tongji Medical College, Huazhong University of Science and Technology, Wuhan, Hubei P.R. China; 2 School of Nursing, Tongji Medical College, Huazhong University of Science and Technology, Wuhan, Hubei P.R. China

**Keywords:** Sexual health, Quality of life, EORTC QLQ-SH22, Simplified Chinese, Cross-cultural adaptation, Psychometric validation

## Abstract

**Background:**

Sexual health is a critical aspect of quality of life in cancer patients. The EORTC QLQ-SH22 (SH22) assesses sexual health–related quality of life (SH-QoL), but no Simplified Chinese version has been validated. This study aimed to translate and culturally adapt the SH22 into Simplified Chinese (SH22-C) and to evaluate its psychometric properties in mainland Chinese cancer patients.

**Methods:**

This study comprised two phases. Phase I involved translation and cultural adaptation of the SH22, following the standardized EORTC translation procedure to ensure clarity and cultural relevance. Phase II was a cross-sectional survey in outpatient oncology clinics of a tertiary hospital in mainland China, with 270 adults completing the SH22-C and 30 participants completing a retest after 2 weeks. Psychometric properties assessed included internal consistency, test–retest reliability, structural validity, and convergent/discriminant validity.

**Results:**

Cognitive interviews led to revisions of ambiguous sexual activity terms, with no further changes in the second round. Phase II demonstrated excellent internal consistency (*Sexual Satisfaction* α = 0.93; *Sexual Pain* α = 0.81) and good test–retest reliability (intraclass correlation coefficients = 0.86 and 0.84, respectively; single-item *r* = 0.73–0.88). Confirmatory factor analysis confirmed a two-factor structure with acceptable fit and factor loadings of 0.62–0.92. Strong convergent validity and low inter-factor correlation (*r* = − 0.26) supported discriminant validity.

**Conclusions:**

The SH22-C showed good cultural equivalence, reliability, and validity in mainland Chinese cancer patients and provides a valid and practical tool for assessing SH-QoL.

**Supplementary Information:**

The online version contains supplementary material available at https://doi.org/10.1186/s12955-026-02567-z.

## Background

Sexual health is a multidimensional construct defined by the World Health Organization as physical, emotional, mental, and social well-being in relation to sexuality [1]. Cancer and its treatments can impair multiple domains of sexual health, including sexual function, body image, and intimate relationships [2], with sexual dysfunction affecting up to 40%–85% of male and 90% of female patients [3, 4]. In China, this issue is further amplified by the growing cancer burden. GLOBOCAN 2022 indicates that China contributes substantially to global cancer incidence and mortality, with age-standardized rates of 209.6 and 127.5 per 100,000 in men and 197.0 and 67.8 per 100,000 in women, respectively [5].With improving survival, the population of cancer survivors is rapidly expanding, shifting the focus of oncology care from survival to long-term quality of life (QoL) [4, 6]. However, sexual health remains a frequently overlooked aspect of QoL in both research and clinical practice [7].

Although American Society of Clinical Oncology (ASCO) and Cancer Care Ontario recommend the proactive assessment and management of sexual health concerns throughout cancer treatment and survivorship [6], such concerns are rarely discussed or systematically assessed in routine oncology practice [8]. This implementation gap is partly attributed to contextual barriers and the lack of standardized, cancer-specific instruments integrated into QoL frameworks [9, 10]. Given that sexual health in cancer patients extends beyond sexual function to include body image, intimacy, emotional well-being, and interpersonal relationships [11], assessment within a QoL framework is essential for capturing its full impact [2, 11]. However, commonly used instruments such as the Female Sexual Function Index (FSFI) [12] and the International Index of Erectile Function (IIEF) [13] primarily assess general sexual functioning and do not capture cancer-specific sexual health concerns, nor are they integrated into the European Organization for Research and Treatment of Cancer (EORTC) QoL framework [14, 15]. In response, the EORTC Quality of Life Group developed the EORTC QLQ-SH22 as a cancer-specific patient-reported outcome measure to assess sexual health-related quality of life in patients with cancer and survivors [2, 16]. Developed in accordance with EORTC guidelines, the SH22 reflects a multidimensional conceptualization of sexual health encompassing physical, psychosexual, and social domains [16]. It includes 22 items, comprising two multi-item scales (Sexual Satisfaction and Sexual Pain) and multiple single, conditional, and gender-specific items [2]. Although the SH22 has been translated into several languages and has shown consistent psychometric performance across validation studies, a validated Simplified Chinese version remains unavailable [2, 16].

Cultural constraints in mainland China often hinder discussions of sexual health [17]. Consequently, sexual dysfunction among Chinese cancer patients is likely underreported due to stigma, limited clinician training, and the absence of culturally appropriate assessment tools [18]. Given the large population of cancer survivors in China, rigorous translation and psychometric validation of the SH22 are essential for accurate identification of unmet sexual health needs in clinical settings and for advancing SH-QoL research in Chinese populations. Therefore, this study aimed to translate and culturally adapt the EORTC QLQ-SH22 into Simplified Chinese following the EORTC translation procedure [19], and to evaluate its structural validity, internal consistency, and test–retest reliability in a sample of Chinese cancer patients in accordance with the Consensus-based Standards for the selection of Health Measurement Instruments (COSMIN) recommendations [20], thereby assessing its applicability in this population.

## Methods

### Study design

**Fig. 1:**
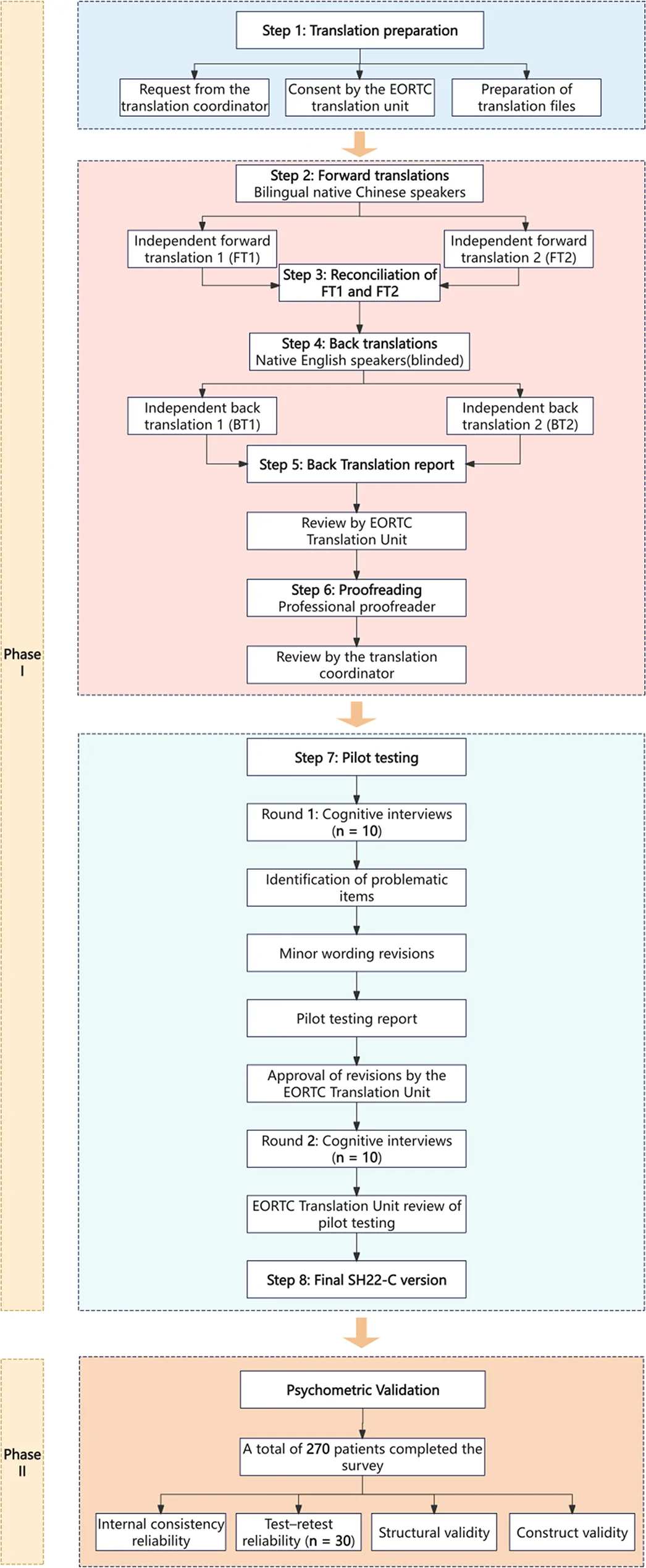
Study flowchart of the translation, cultural adaptation, and psychometric validation of the SH22-C

This study was conducted in two sequential phases. In Phase 1, the Simplified Chinese version of the EORTC QLQ-SH22 (SH22-C) was developed in accordance with the standardized translation procedure of the EORTC QoL Group (Fourth Edition, 2017) [19]. This multistep process was designed to ensure conceptual equivalence, linguistic accuracy, and cultural appropriateness. In Phase 2, the translated SH22-C was administered to a clinical sample to evaluate its psychometric validity and reliability. The study flowchart is presented in Fig. 1.

### Phase I: Translation and cultural adaptation

First, formal permission for translation was obtained from the EORTC Translation Unit (TU), which subsequently provided the original English version of the SH22 and relevant reference materials. Two bilingual native Chinese medical professionals independently produced forward translations. These translations were then reconciled by a third native Chinese translator, who synthesized the two versions into a single consensus translation and documented all decisions made during reconciliation.

Two independent back-translations were subsequently conducted by translators fluent in both Chinese and English. Neither translator had participated in the forward translation, and both were blinded to the original SH22. Each translator independently retranslated the reconciled Simplified Chinese version back into English, allowing the TU to compare the back-translated versions with the original and assess semantic fidelity.

After several rounds of review and clarification with the TU, the reconciled Chinese version underwent professional proofreading by a certified translator to ensure clarity, consistency, and grammatical accuracy. Following proofreading, two rounds of cognitive debriefing (*n* = 10 per round; *N* = 20) were conducted with native Chinese-speaking cancer patients purposively sampled to reflect diverse demographic and clinical characteristics, such as age, gender, education level, and cancer type. Participants completed the translated SH22-Chinese Version (SH22-C) and were interviewed using a predefined EORTC patient response form to assess item clarity, comprehensibility, and cultural relevance. Patients were encouraged to identify items that were difficult, confusing, upsetting, or culturally inappropriate, and to offer paraphrased alternatives.

All feedback was summarized in a pilot-testing report and submitted to the TU for further review. Minor revisions were incorporated based on patient feedback and TU recommendations. The TU then granted final approval of the SH22-C. Details of the translation and cultural adaptation process are provided in Additional file 1, and the finalized Simplified Chinese version of the SH22 questionnaire is presented in Additional file 2.

### Phase II: Psychometric validation in a clinical sample

#### Participants

This cross-sectional study was conducted in the outpatient oncology clinics of a tertiary teaching hospital in mainland China. Eligible participants met the following criteria: (1) Adults aged ≥ 18 years; (2) Histologically confirmed malignancy; (3) Awareness of their cancer diagnosis; (4) Ability to communicate in Simplified Chinese, and capable of completing self-administered questionnaires; (5) No documented cognitive impairment, defined as a clinically diagnosed neurocognitive disorder recorded in the medical record (e.g., dementia or intellectual disability) that could interfere with questionnaire comprehension and completion; (6) A history of partnered sexual activity, as required by the SH22, which includes partner-related and conditional items to ensure item applicability and response validity in accordance with its original framework. This criterion was assessed during recruitment using a standardized, self-administered written screening question. Participants completed the screening privately in a designated area and recorded their responses without verbal disclosure. When necessary, trained research staff provided brief clarification in a low voice to maintain privacy. Patients were excluded if they had serious visual, hearing or physical impairments that hindered independent completion of the questionnaire; or were experiencing an acute medical emergency or clinical instability during recruitment.

The required sample size was estimated based on psychometric research standards for confirmatory factor analysis (CFA). As the EORTC QLQ-SH22 is an established instrument developed by the EORTC QoL Group without item modification, the recommended ratio of 5–10 participants per item was adopted [21]. For the 22-item SH22, the minimum required sample ranged from 110 to 220 participants. A total of 270 patients were ultimately enrolled.

### Data collection

Eligible patients were identified by the clinical team and approached consecutively during routine outpatient visits. After receiving a brief explanation of the study, those who agreed to participate provided written informed consent. Participants then completed the Simplified Chinese version of the EORTC QLQ-SH22 (SH22-C) independently in a private space within the clinic. Data were collected using an electronic questionnaire administered via a secure online survey platform (Wenjuanxing), accessed via on-site QR code scanning, with built-in settings requiring item completion prior to submission to minimize item-level missing data. When necessary, trained research staff were available to clarify instructions and answer procedural questions, but no assistance was provided with item interpretation or response selection. Sociodemographic information was collected using a structured self-report form, and relevant clinical data were obtained from medical records with participants’ consent. To examine test–retest reliability, a subsample of 30 participants completed the SH22-C again 2 weeks after the initial assessment under the same administration procedures.

### Measures

#### Sociodemographic and clinical information

Sociodemographic information was obtained by patient self-report using a structured questionnaire and included age, gender, marital status, place of residence, occupation, education level, and monthly household income per capita. Clinical information was extracted from medical records and included cancer diagnosis, cancer stage and treatment status.

#### EORTC QLQ-SH22-C

The EORTC QLQ-SH22 is a cancer-specific instrument designed to assess sexual health–related QoL [2, 16]. It comprises 22 items, including two multi-item scales—Sexual Satisfaction (8 items: 5 core + 3 conditional) and sexual pain (3 items: 2 core + 1 conditional), as well as 11 single-item indicators covering importance of sexual activity, decreased libido, worry incontinence, fatigue, treatment effect on sexual activity, communication with professionals, insecurity with the partner, confidence erection, body image (male), body image (female), vaginal dryness [2]. All items are rated on a four-point Likert scale ranging from 1 (“Not at all”) to 4 (“Very much”) [19].

The Simplified Chinese version (EORTC QLQ-SH22-C) used in this study was developed following the standardized EORTC translation procedure and retains the same conceptual framework, item structure, response options, and scoring algorithm as the original instrument [19]. Only minor wording refinements were introduced to improve semantic clarity and cultural appropriateness for mainland Chinese patients, without altering the underlying constructs. Scores were calculated according to the EORTC scoring manual. Multi-item scale scores were calculated as the mean of the corresponding item responses and linearly transformed to a 0–100 scale, with higher scores indicating greater sexual symptom burden or problems. Reverse scoring was performed for selected items (Items 1, 3, 4, 9, 10, 12, 14, 17, 18, 19, and 21) before transformation, and conditional items were scored only when relevant [22].

### Statistical analysis

All statistical analyses were performed using IBM^®^ SPSS Statistics version 27.0 and AMOS version 28.0 (IBM Corp., Armonk, NY, USA). Descriptive statistics, including means, standard deviations, frequencies, and percentages, were used to summarize participants’ sociodemographic and clinical characteristics. Before psychometric analyses, data completeness and the eligibility of responses for each analysis were reviewed. Cronbach’s α was calculated only for multi-item scales, test–retest reliability was assessed only among clinically stable participants who completed both the baseline and retest assessments, and CFA was performed to evaluate the prespecified two-factor structure of the SH22-C using maximum likelihood estimation. Because the electronic questionnaire required item completion before submission, no item-level imputation was performed; participants who did not submit the questionnaire were excluded from the final analytic sample.

First, **internal consistency reliability** was evaluated using Cronbach’s α coefficients for multi-item scales of the SH22-C, with α ≥ 0.70 indicating acceptable reliability [23]. **Test-retest reliability** was assessed in a subset of 30 clinically stable participants who completed the SH22-C again approximately two weeks after the initial assessment, using the same administration procedures, with a research nurse available only for clarification if needed. Temporal stability was quantified using intraclass correlation coefficients (ICCs) with 95% confidence interval for multi-item scales and Pearson’s correlation coefficients (r) for single-item measures [24, 25], with ICCs interpreted as poor (< 0.50), moderate (0.50 to < 0.75), good (0.75 to < 0.90), or excellent (≥ 0.90) reliability [26].

Second, **structural validity** was examined through CFA using the maximum likelihood estimation in AMOS. Model fit was assessed using multiple indices: chi-square to degrees of freedom ratio (χ²/df), root mean square error of approximation (RMSEA), comparative fit index (CFI), and Tucker–Lewis index (TLI) [27]. Model fit was considered acceptable when χ²/df < 3, RMSEA < 0.08, and CFI and TLI > 0.90 [27]. Standardized factor loadings were examined to assess the contribution of each item to its corresponding latent construct, with loadings ≥ 0.50 considered acceptable and ≥ 0.60 considered indicative of good item–factor relationships [28]. Additionally, **convergent and discriminant validity** were evaluated using composite reliability (CR) and average variance extracted (AVE) [29]. Convergent validity was deemed adequate when CR ≥ 0.70 and AVE ≥ 0.50 [30], while discriminant validity was supported when inter-factor correlations were < 0.85 and the square root of each construct’s AVE exceeded its correlations with other constructs [30]. All statistical tests were two-tailed, and a *p* value < 0.05 was considered statistically significant.

### Ethical statement

This study was approved by the Medical Ethics Committee of Tongji Hospital, Tongji Medical College, Huazhong University of Science and Technology (Approval No. TJ-IRB202407137). Written informed consent was obtained from all participants prior to enrollment. The study adhered to institutional and national ethical guidelines and the principles of the Declaration of Helsinki (1964) and its later amendments. All data were anonymized to ensure confidentiality.

## Results

### Phase I: Translation and cultural adaptation of the SH22-C

The SH22-C was developed in accordance with the official EORTC translation guidelines and underwent two consecutive rounds of pilot testing with cognitive interviews. In Phase I, 23 patients were approached for cognitive debriefing, including 12 in round 1 and 11 in round 2. Ten participants completed the cognitive interviews in each round, yielding a total of 20 completed interviews. The characteristics of participants in rounds 1 and 2 are presented separately in Table 1.

**Table 1:** Participant characteristics in rounds 1 and 2 of the cognitive interviews (*N* = 20)

Variable	Round 1 (*n* = 10)	Round 2 (*n* = 10)
**Gender**		
Male	5	5
Female	5	5
**Age (years)**		
<60	5	5
≥ 60	5	5
**Tumor site**		
Breast	1	2
Gynecologic	2	1
Digestive system	1	2
Respiratory system	2	1
Urinary system	2	1
Hematologic/lymphatic	1	1
Male genital	1	2
**Cancer Stage**		
I	3	3
II	3	3
III	2	2
IV	2	2
**Treatment Status**		
Active Treatment	6	6
Survivorship	4	4
**Marital status**		
Unmarried	1	1
Married	7	8
Divorced	1	1
Widowed	1	0
**Place of residence**		
Urban	6	6
Rural	4	4
**Occupation**		
Employed	4	5
Unemployed	6	5
**Education level**		
Primary school or below	2	1
Junior high school	2	3
Senior high school/technical secondary	2	2
College or undergraduate	3	2
Master’s degree or above	1	2

### First-round pilot testing

Cognitive interviews were conducted with 10 cancer patients to assess the clarity, comprehensibility, and cultural appropriateness. Several patients reported difficulties in interpreting specific expressions, particularly in items related to sexual activity (e.g., Q1 “an active sex life,” Q17 “sexually active,”). Based on their feedback, minor wording modifications were made to enhance semantic clarity and cultural acceptability. For instance, the item “How important to you is an active sex life?” was revised to “How important is sex life to you?” and “Have you been sexually active?” was rephrased as “Have you had a sex life?”. For Item 11, minor wording refinement was made in the Simplified Chinese version based on patient feedback to improve readability while preserving the original meaning. All item-level revisions were reviewed and approved by the EORTC. Detailed feedback and corresponding decisions from the first round of pilot testing are summarized in Table 2.

**Table 2:** Cognitive interview feedback from the first round of pilot testing (*n* = 10)

Item No.	Item content	Patient feedback	Decision and EORTC approval
1	How important to you is an active sex life?	Six participants reported difficulty understanding the term “active,” particularly regarding its intended meaning related to sexual frequency.	Removal of the term “active” was approved by the EORTC.
11	Have you been worried that your partner may cause you pain during sexual contact?	Six participants indicated that the Chinese wording was relatively lengthy and suggested simplification to improve readability.	The Simplified Chinese wording was refined for clarity while preserving the original meaning, as approved by the EORTC.
17	Have you been sexually active?	Six participants reported that the term “active” was unclear and difficult to interpret in the context of sexual frequency.	Removal of the term “active” was approved by the EORTC.

### Second-round pilot testing and approval

Additional 10 cancer patients completed cognitive interviews using the revised version. All participants reported that the questionnaire instructions and items were clear, understandable, and culturally acceptable, and no further modifications were suggested. The EORTC TU reviewed the complete documentation, including pilot-testing results and the rationale for all wording changes. Following this review, the final version of the SH22-C was approved for use in the psychometric validation phase.

### Observed response patterns and cultural considerations

During the cognitive interviews, several participants expressed hesitation or discomfort when responding to items explicitly addressing sexual activity or communication about sexual problems with partners or healthcare professionals. Some participants indicated that they had never previously discussed sexual concerns in a clinical context, and a small number questioned whether it was appropriate to answer such questions in the presence of family members.

In addition, items related to sexual activity and partner communication exhibited slightly higher proportions of missing or “not applicable” responses compared with other items, particularly among older and unmarried participants. These findings indicate that cultural norms regarding sexuality, relational status, and privacy may influence response behavior to certain SH22-C items in mainland China.

### Phase II: Psychometric validation of SH22-C

#### Sample characteristics

In Phase II, 305 patients were approached during routine outpatient visits. Of these, 285 met the eligibility criteria and provided informed consent. Fifteen participants who initially consented did not submit the questionnaire, primarily due to discomfort with the sensitive nature of the questions. Ultimately, 270 cancer patients completed the baseline SH22-C and were included in the psychometric validation analyses, with a completion rate of 88.5%.

Participants ranged in age from 20 to 80 years, with a mean age of 51.3 ± 11.9 years. The sample included 176 females (65.2%) and 94 males (34.8%). The most represented age group was 51–65 years (*n* = 117, 43.3%), and breast cancer accounted for the largest proportion of tumors (*n* = 112, 41.5%). Most participants were married (*n* = 233, 86.3%), and 124 (45.9%) were employed. Detailed information of participant characteristics is displayed in Table 3.

**Table 3:** Sociodemographic and clinical characteristics of participants in Phase II (*n* = 270)

Variable	*n* (%) / Mean ± SD
**Gender**	
Male	94 (34.8)
Female	176 (65.2)
**Age (years)**	51.3 ± 11.9 (range: 20–80)
**Age group (years)**	
20–35	31 (11.5)
36–50	94 (34.8)
51–65	117 (43.3)
66–80	28 (10.4)
**Tumor site**	
Breast	112 (41.5)
Gynecologic	52 (19.3)
Digestive system	61 (22.6)
Respiratory system	17 (6.3)
Head and neck	17 (6.3)
Urinary system	5 (1.9)
Hematologic/lymphatic	2 (0.7)
Male genital	2 (0.7)
Bone/soft tissue	2 (0.7)
**Cancer Stage**	
I	105 (38.9)
II	64 (23.7)
III	59 (21.9)
IV	42 (15.6)
**Treatment Status**	
Active Treatment	187 (69.3)
Survivorship	83 (30.7)
**Marital status**	
Unmarried	18 (6.7)
Married	233 (86.3)
Divorced	15 (5.6)
Widowed	4 (1.5)
**Place of residence**	
Urban	177 (65.6)
Rural	93 (34.4)
**Occupation**	
Employed	124 (45.9)
Unemployed	146 (54.1)
**Education level**	
Primary school or below	23 (8.5)
Junior high school	90 (33.3)
Senior high school/technical secondary	51 (18.9)
College or undergraduate	95 (35.2)
Master’s degree or above	11 (4.1)
**Monthly household income per capita (USD)**	
< $420	80 (29.6)
$420–$700	92 (34.1)
$700–$1390	81 (30.0)
> $1390	17 (6.3)

### Reliability

The SH22-C demonstrated satisfactory reliability in terms of both internal consistency and temporal stability. The *Sexual Satisfaction* scale (8 items: five core and three conditional) showed excellent internal consistency (Cronbach’s α = 0.93), while the *Sexual Pain* scale (3 items: two core and one conditional) exhibited good reliability (Cronbach’s α = 0.81). Test-retest reliability was likewise acceptable to excellent, with ICCs indicating good stability over time for the multi-item scales (*Sexual Satisfaction*: ICC = 0.86, 95% CI: 0.70–0.94; *Sexual Pain*: ICC = 0.84, 95% CI: 0.67–0.93). For the single-item measures, temporal stability assessed by Pearson’s correlation coefficients was moderate to high (*r* = 0.73–0.88). Detailed reliability indices for each scale and item are presented in Table 4.

**Table 4:** Descriptive statistics and reliability indices of the SH22-C

Scale	Item Number	Mean ± SD	ICC (95% CI) / *r*	CR	AVE	Cronbach’s α
Sexual Satisfaction	8	2.03 ± 0.62	0.86 (95% CI: 0.70–0.94)	0.92	0.61	0.93
Sexual Pain	3	1.61 ± 0.51	0.84 (95% CI: 0.67–0.93)	0.83	0.62	0.81
Sexual activity	1	1.36 ± 0.58	0.83	N.A.		
Decreased Libido	1	2.08 ± 1.04	0.81			
Incontinence	1	1.45 ± 0.58	0.76			
Fatigue	1	1.83 ± 0.78	0.74			
Treatment	1	1.82 ± 0.82	0.79			
Communication with professionals	1	1.17 ± 0.42	0.78			
Partnership	1	1.52 ± 0.67	0.86			
Confidence Erection	1	2.03 ± 0.83	0.73			
Body image (male)	1	1.81 ± 0.78	0.79			
Body image (female)	1	1.85 ± 0.90	0.88			

Notes. Cronbach’s α was calculated for multi-item scales only. Test–retest reliability was assessed using ICC for multi-item scales and r for single-item scales. CI = confidence interval; CR = composite reliability; AVE = average variance extracted. N.A. = not applicable

### Structural validity

**Fig. 2:**
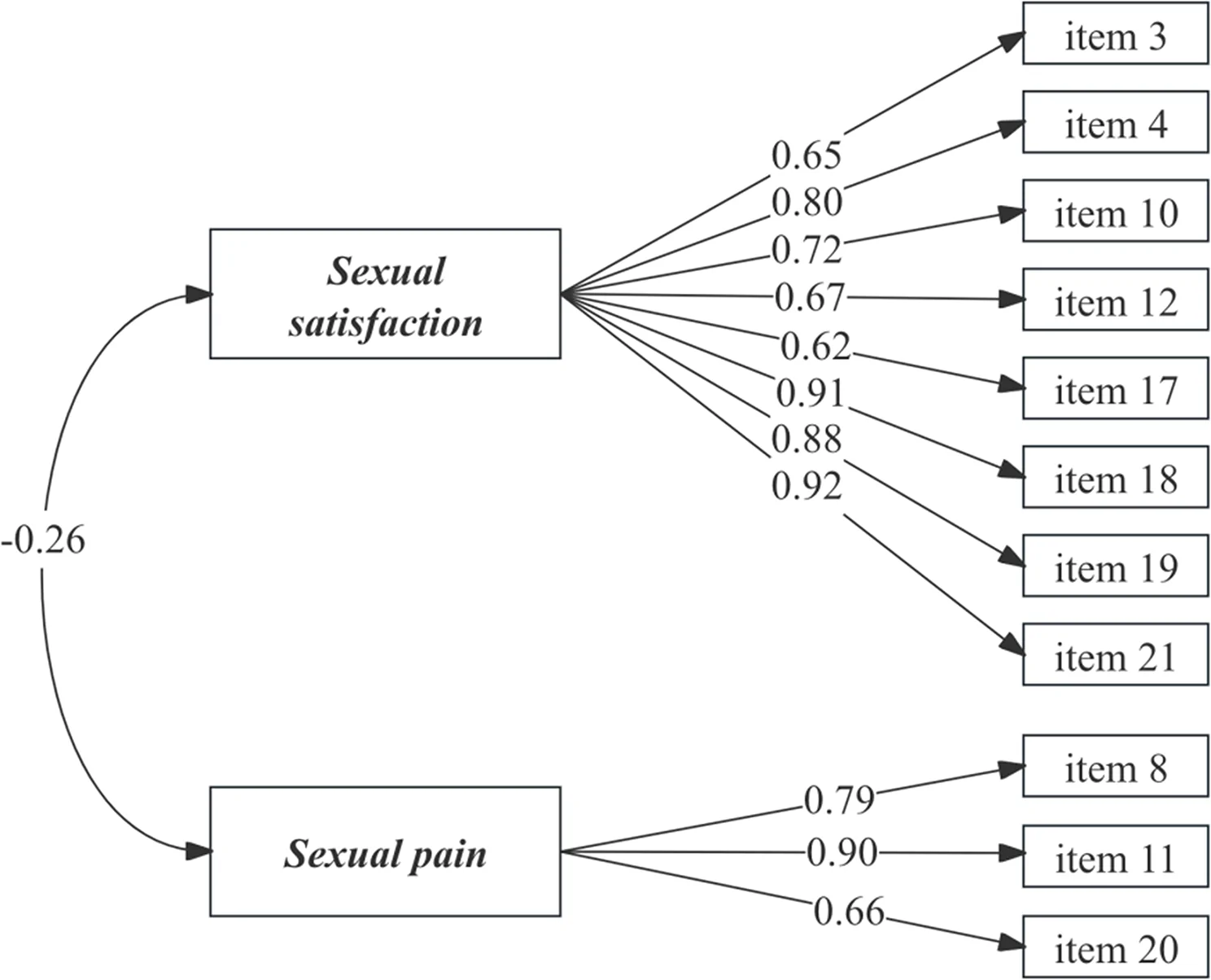
Confirmatory factor analysis (CFA) model of the SH22-C

Structural validity of the SH22-C was examined using CFA. The hypothesized two-factor model (*Sexual Satisfaction* and *Sexual Pain*) showed an acceptable fit to the data (χ²/df = 2.64, RMSEA = 0.078, CFI = 0.946, TLI = 0.918). Standardized factor loadings for the 11 items ranged from 0.62 to 0.92, supporting the adequacy of the factor structure (Fig. 2).

### Convergent and discriminant validity

The SH22-C demonstrated satisfactory convergent validity, with CR and AVE values of 0.92 and 0.61 for *Sexual Satisfaction*, and 0.83 and 0.62 for *Sexual Pain*. The correlation between the two latent factors was *r* = − 0.26 (|r| < 0.85), the square root of the AVE for each construct was greater than its correlation with the other construct, supporting adequate discriminant validity.

## Discussion

This study translated and culturally adapted the EORTC QLQ-SH22 into Simplified Chinese, followed by pilot testing and comprehensive psychometric validation. Overall, the SH22-C demonstrated satisfactory psychometric performance and cultural acceptability for assessing sexual health–related quality of life among Chinese cancer patients.

### Translation and cultural adaptation of the SH22-C

Consistent with other EORTC translation projects [31], the EORTC QLQ-SH22 was translated and culturally adapted into Simplified Chinese using standardized procedures emphasizing conceptual equivalence [19]. Cognitive interviews identified linguistic ambiguities in items related to sexual activity, particularly “active sex life” and “sexually active,” which were commonly interpreted as sexual frequency rather than the presence of sexual activity in mainland China, potentially introducing measurement bias [32, 33]. Minor wording refinements were made to enhance clarity while preserving the original meaning [19], and the item on pain during sexual activity was streamlined for readability without altering its conceptual content [19].

Participants’ hesitation in responding to sexually explicit items reflects the cultural sensitivity surrounding sexual health in mainland China [18], consistent with previous studies highlighting the influence of traditional cultural values on responses to sexual health measures [34]. In this context, sexuality is often regarded as a private and sensitive topic with limited open discussion in clinical or research settings, particularly among older adults and individuals with more conservative attitudes. Consistently, the relatively older age profile of the sample (with a substantial proportion aged over 50 years) may have further contributed to cautious responses to items related to sexual activity, intimacy, and partner communication. Accordingly, in similar cultural contexts, greater attention should be given to privacy-protective administration and culturally sensitive communication strategies to enhance disclosure and improve identification of unmet sexual health needs. Private administration and brief normalization statements may help improve response accuracy, alongside optional follow-up counseling or referral pathways [35]. Implementation strategies that reduce stigma are particularly important in China, where clinicians may feel less confident initiating sexual health discussions [18]. Future research should further evaluate culturally tailored implementation approaches and identify subgroups at risk of underreporting.

### Reliability of the SH22-C

In this study, the SH22-C demonstrated good to excellent reliability, with internal consistency suitable for group-level comparisons [23]. Test-retest reliability over two weeks showed temporal stability, with strong ICCs for multi-item scales and adequate Pearson correlations for single-item measures [20, 25, 26]. These findings align with the original validation, where Sexual Satisfaction and Sexual Pain scales demonstrated good internal consistency (α = 0.90 and 0.80) and acceptable fit across 13 countries [2] as well as with Moroccan Arabic and Turkish versions (α = 0.79–0.91; CFI ≈ 0.98–0.99) [36, 37]. Thus, our results confirm that the Simplified Chinese version maintains the core psychometric properties of the original SH22.

### Validity of the SH22-C

Structural validity analyses supported the prespecified measurement model of the SH22-C for its two multi-item scales, *Sexual Satisfaction* and *Sexual Pain*, in line with the original EORTC QLQ-SH22 framework [2]. CFA of the 11 items supported the proposed two-factor structure, with acceptable model fit and standardized factor loadings indicating that all items contributed meaningfully to their respective latent constructs [27, 30]. Interestingly, we observed slightly lower loadings for items on emotional comfort and partner communication, which may reflect cultural conservatism regarding sexual discussion in China [38]. Compared with recent adaptations, our model fit was slightly lower than that reported for the Moroccan Arabic (RMSEA = 0.04; CFI/TLI = 0.99/0.99) and Turkish versions (CFI/TLI = 0.99/0.99; RMSEA = 0.04) [36, 37], suggesting possible cross-sample or cross-cultural differences in item performance.

Consistent with the original EORTC QLQ-SH22 and other language adaptations, the SH22-C multi-item scales demonstrated adequate convergent validity [2, 30, 37]. Discriminant validity was supported by a weak negative correlation between Sexual Satisfaction and Sexual Pain, in line with prior findings and confirming construct distinction [2, 29, 37]. Overall, the SH22-C retained the structural integrity and construct equivalence of the original instrument within the Chinese context.

### Strengths and limitations

This study is the first to translate and validate the EORTC QLQ-SH22 in Simplified Chinese for use among Chinese cancer patients, following standardized EORTC translation procedures and applying rigorous psychometric testing to ensure methodological robustness.

However, several limitations should be acknowledged. The single-center design and heterogeneous sample with a relatively older age distribution, together with reliance on self-reported data and the presence of conditional or gender-specific items, may limit external validity and introduce response bias. In addition, the use of an electronic survey platform requiring complete responses prior to submission minimized item-level missing data but may have introduced selection bias, as participants who were unwilling or unable to complete all items were not included in the final analytic sample. These factors may restrict generalizability to broader and younger populations, contribute to underreporting of sexual health problems, and necessitate caution when interpreting prevalence estimates and applying the SH22-C across different subgroups and clinical contexts. Moreover, the cross-sectional design limits the evaluation of responsiveness and longitudinal stability of the SH22-C. Therefore, the instrument should be primarily used for cross-sectional assessment, and caution is needed when applying it to monitor changes over time in longitudinal or interventional studies. Finally, although convergent validity was evaluated using CR and AVE within the CFA framework, convergent or criterion validity against external sexual health or quality-of-life instruments was not assessed. This limits cross-instrument comparability and suggests that findings should be interpreted cautiously when comparing results across studies or instruments. Future research is needed to conduct multicenter and longitudinal validation as well as cross-instrument comparisons.

## Conclusions

The Simplified Chinese version of the EORTC QLQ-SH22 (SH22-C) demonstrated satisfactory reliability, validity, and cultural equivalence among Chinese cancer patients. The two-factor structure of *Sexual Satisfaction* and *Sexual Pain* showed strong internal consistency and acceptable model fit. The SH22-C provides a practical and culturally appropriate tool for assessing sexual health-related quality of life in both clinical and research settings. Further studies are needed to confirm its longitudinal stability and responsiveness across different cancer populations.

## Supplementary Information

Below is the link to the electronic supplementary material.

Supplementary Material 1

Supplementary Material 2

## Data Availability

The datasets generated and/or analyzed during the current study are not publicly available due to patient privacy and institutional regulations, but are available from the corresponding author on reasonable request.

## Abbreviations

EORTC: European Organization for Research and Treatment of Cancer
QLQ-SH22: EORTC Quality of Life Questionnaire–Sexual Health 22
SH22-C: Simplified Chinese version of the EORTC QLQ-SH22
PRO: Patient-reported outcome
CFA: Confirmatory factor analysis
CR: Composite reliability
AVE: Average variance extracted
ICC: Intraclass correlation coefficient
RMSEA: Root mean square error of approximation
CFI: Comparative fit index
TLI: Tucker–Lewis index
