# Supplementary material for: Translation, cultural adaptation, and psychometric validation of the simplified Chinese EORTC QLQ-SH22 among cancer patients
Source: Health Qual Life Outcomes. 2026 Jun 18;24:112. doi: 10.1186/s12955-026-02567-z (PMC13548554; doi:10.1186/s12955-026-02567-z)
Supplement: Supplementary file 1 [file 12955_2026_2567_MOESM2_ESM.pdf]

Additional file 2. Availability statement for the finalized SH22-C questionnaire

The detailed translation and cultural adaptation process, including forward translation, back translation, expert review, and pilot testing, is provided in Additional file 1.

As the SH22-C is the final Simplified Chinese version evaluated in this study, and the EORTC QLQ-SH22 is a copyrighted instrument, the full questionnaire is not reproduced in the supplementary files. Access to the questionnaire should be requested through the EORTC Quality of Life Group in accordance with their conditions of use. Further information is available from the official website of the European Organisation for Research and Treatment of Cancer (EORTC): https://www.eortc.org/
